# The promising role of Gelsolin expression to predict survival in patients with squamous cell carcinoma of the larynx

**DOI:** 10.1016/j.bjorl.2021.05.009

**Published:** 2021-06-03

**Authors:** Akın Şahin, Necati Enver, Selim Yiğit Erçetin, Zeliha Leyla Cinel, Abdullah Çağlar Batman

**Affiliations:** a Marmara University School of Medicine, Department of Otorhinolaryngology, Istanbul, Turkey; b Marmara University School of Medicine, Department of Pathology, Istanbul, Turkey

**Keywords:** Gelsolin, Immunohistochemistry, Laryngeal squamous cell carcinoma, Biomarker, Prognosis

## Abstract

**Introduction:**

Gelsolin protein has important cellular functions, including cell motility and apoptosis. Altered gelsolin expression has been reported in several types of neoplasms, but clinicopathological features of gelsolin are currently unclear in patients with laryngeal squamous cell carcinoma.

**Objectives:**

Our aim is to investigate the clinicopathological significance of gelsolin as a prognostic biomarker for laryngeal squamous cell carcinoma.

**Methods:**

Tissue specimens from 168 patients with laryngeal squamous cell carcinoma were immunohistochemically assessed for the Gelsolin expression. Prognostic significance of Gelsolin and its interaction with clinical parameters was analysed.

**Results:**

Gelsolin expression was confirmed in 70.2% of cases. Gelsolin expression is significantly associated with tumor stage, tumor grade, and locoregional recurrence. Kaplan–Meier survival curves revealed that Gelsolin expression inversely correlated with both disease-specific and overall survival.

**Conclusion:**

This research is the first to demonstrate that Gelsolin expression is associated with a poor prognosis in laryngeal squamous cell carcinoma. Gelsolin is a novel promising biomarker and attractive target for the treatment of laryngeal squamous cell carcinoma.

## Highlights

• Gelsolin overexpression significantly correlated with unfavorable survival.
• Increased gelsolin expression was associated with lymphovascular invasion.
• Gelsolin expression was higher in patients with advanced-stage laryngeal carcinoma.
• Locoregional recurrence risk is higher in cases wıth gelsoline overexpression.
• Gelsolin expression could be regarded as a novel independent prognostic biomarker.

## Introduction

Cancer of the larynx is the second most common cancer among head and neck neoplasms, after oral cavity cancers. The incidence of laryngeal cancer is 2.7/100,000 and more than 200,000 new cases are seen worldwide annually.^1^ Although various histological types of malignant cases can be seen in the larynx, squamous cell carcinomas (SCC) include about 95% of laryngeal cancer cases.^2^ Over the past few decades, the incidence of laryngeal SCC, or LSCC, has increased in in South-East Asia and Western Pacific and gradually declined in Europe, presumably due to differences in alcohol and tobacco consumption.^1^, ^3^

Although several factors relate to LSCC prognosis, TNM stage, histopathological grade, and primary tumor site are the most important factors among them.^4^, ^5^ Even so, these factors can provide useful information about prognosis and survival; however, they can sometimes be inadequate due to the fact that host- and tumor-related factors affect them. In order to have better tools for prognosis and survival, intracellular molecular pathways in larynx carcinogenesis must be revealed. Therefore, the discovery and validity of novel biomarkers that play a role in carcinogenesis of the larynx will enable the development of more effective laryngeal cancer treatment modalities.^6^

Gelsolin is a 82-kDa actin regulator protein that plays a key role in cytoskeleton organization, cell motility, cell growth, and apoptosis.^7^ Gelsolin has the capability of capping and severing actin molecules, thus modulating polymerization and length of filaments. Free Ca^2+^ ions, temperature, Ph, and phosphatidylinositol 4.5 bisphosphate (PIP2) are the main regulatory factors for G-protein. Intracellular interaction of gelsolin and caspase-3 has been shown to accelerate apoptosis-related morphological changes, thus promoting apoptosis. On the other hand, full-length gelsolin induced by agents such as ceramide, dexamethasone, and anti-Fas antibody inhibit apoptosis.^8^ The inhibiting or promoting effect of gelsolin may vary depends upon the type of tissues and cells. Gelsolin upregulation and an adverse effect on prognosis have been reported in lung, prostate, and oral cavity cancers;^9^, ^10^, ^11^ however, decreased expression of gelsolin suggests a tumor-suppressing role in breast and colon malignancies.^12^, ^13^ Regarding the effects of gelsolin on carcinogenesis, currently no studies exists on the expression of gelsolin in LSCC tissues and its impact on prognosis.

In this study, we investigated the expression level of gelsolin using immunohistochemistry (IHC) and evaluated its correlation with clinicopathological and prognostic parameters in LSCC patients. Our aim in this research was to clarify the relationship between gelsolin and laryngeal cancer and to investigate the usability of gelsolin as a novel biomarker to predict prognosis in LSCC.

## Methods

### Patient population and tissue samples

We analyzed 168 patients with pathologically confirmed LSCC who underwent primary open partial or total laryngectomy between January 2010 and December 2019. None of the patients had received either chemotherapy or radiotherapy previously. Before surgery, all patients were investigated by laryngoscopy with laryngeal biopsies, head, and neck contrast-enhanced computed tomography (CT), and/or magnetic resonance imaging (MRI), neck ultrasonography, and chest radiography. Informed consent was obtained from all patients who were living during this study. This research study was approved by the Institutional Review Board (Approval ID: 09.2018.436) and was conducted in accordance with the principles of the Helsinki Declaration.

### Immunohistochemistry and evaluation of staining

Immunohistochemical procedures were conducted in compliance with the manufacturer's instructions using an automated immunohistochemical stainer (Ventana BenchMark ULTRA, Ventana Medical Systems, Inc., Tucson, AZ, USA). Surgical specimens were fixed in neutral buffered formalin and embedded in paraffin wax. The 4-μm-tissue sections were deparaffinized using the EZ Prep solution (Ventana Medical Systems, Inc., Tucson, AZ, USA). A Cell Conditioning-1 solution (Ventana Medical Systems, Inc.) was used for heat-induced antigen retrieval at 70 °C for 60 min. Endogenous peroxidase activity was blocked using hydrogen peroxide and 3.3′-Diaminobenzidine tetrahydrochloride (UltraView™ Universal DAB Detection Kit, Ventana Medical Systems). Tissue sections were then incubated with the monoclonal anti- gelsolin antibody (clone 2C4, Sigma-Aldrich, St. Louis, MO, USA) at a specified dilution. Afterwards, sections were counterstained with hematoxylin and bluing reagent, cleared, and coverslipped with the mounting media.

**Figure 1:**
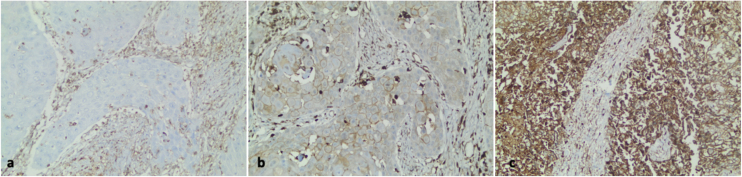
Gelsolin immunohistochemical expression in human laryngeal carcinoma. (a) Negative gelsolin expression in the neoplastic epithelium of laryngeal carcinoma (×200). (b) A case of laryngeal carcinoma with low gelsolin expression (×200). (c) High gelsolin expression in human laryngeal carcinoma (×200).

All stained sections were selected in blind fashion and examined by two pathologists independently who had no prior knowledge of patients’ clinicopathological outcomes. Cells were evaluated according to their cytoplasmic staining levels. A four-scale scoring scheme was used as follows: tumors absent of gelsolin expression were scored 0, weak expressions were scored 1, moderate expressions were scored 2, and strong expressions were scored 3 (Fig. 1). We used the Gelsolin Expression Index achieved by multiplying the percentage of staining tumor cells for each of the three positive scores (1+, 2+, or 3+), as described previously in the literature.^14^, ^15^ The median value was used as the cut-point for the dichotomization of the gelsolin expression due to the skewed index distribution (weak or negative if index ≤30, and strong or positive if index >30).

### Statistical analysis

A *p*-value of < 0.05 was determined as a statistically significant level. Associations between clinicopathological parameters of LSCC patients and IHC score of gelsolin protein were analyzed using the Chi-Square (χ2) test or Fisher's exact test. The Kaplan–Meier method was used to estimate Disease-Free Survival (DFS) and Overall Survival (OS) analysis, and survival differences were analyzed by a log-rank test. Multivariate analysis was determined by the Cox proportional hazards model to identify independent prognostic factors. Statistical analyses were performed using SPSS 20.0 statistical software for Windows (IBM, Chicago, Illinois, USA).

## Results

### Clinicopathologic parameters

This study included 168 patients who were diagnosed with LSCC. Overall average age of patients was 62.23 ± 10.36 with a range of 41–88 years. Patients were predominantly male (158 men, 10 women). More than 90% of the patients were either former smokers (63%) or current smokers (28%), and around 9% of the patients were non- smokers. LSCC was located in the glottis area of 108 (64.2%) patients, compared to 55 (32.9%) patients in the supraglottic region. Tumors were staged based on the eighth edition of the American Joint Committee on Cancer. Forty-one percent of patients were in T1 to T2 stage, 30.0% in T3, and 29% in T4. Fifty-five patients out of 168 (32.7%) with LSCC had positive lymph nodes. Sixty-four patients (38%) were classified with early-stage laryngeal cancer, while 104 patients (62%) were classified with advanced-stage laryngeal cancer. Median duration of followup was 49.2 months (3–120 months). Recurrences were observed in 60 of 168 (35.7%) cases.

### Gelsolin expression in LSCC and its clinicopathological features

**Figure 2:**
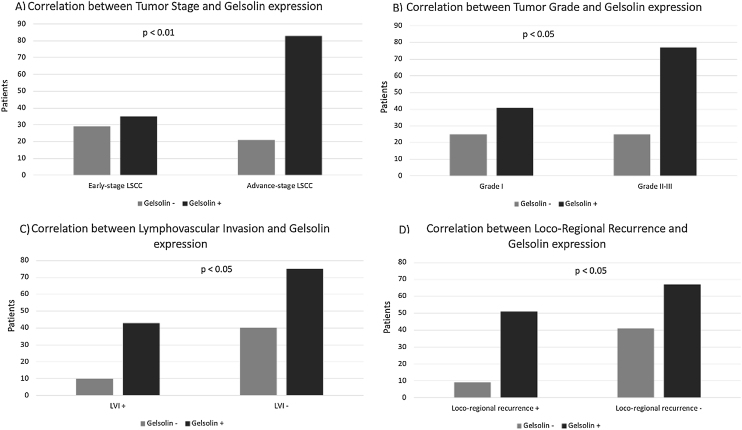
Relationship of gelsolin expression and clinicopatological features. gelsolin expression correlated with tumor stage (A), tumor grade (B), lymphovascular invasion (C), loco-regional recurrence (D).

Analysis of protein expression using IHC was carried out for gelsolin in all samples. Immunostaining showed that gelsolin had positive expression in 118 (70.2%) of 168 human LSCC samples. There was a strong association between expression of gelsolin and tumor stage, tumor differentiation, tumor recurrence, and Lymphovascular Invasion (LVI), with *p*-values of 0.001, 0.031, 0.018 and 0.016 respectively (Fig. 2). Conversely, no significant correlation was identified between gelsolin protein expression and age, sex, lymph node status, perineural invasion (PNI), and primary location of laryngeal carcinoma, with *p*-values of 0.794, 0.937, 0.326 and 0.872 respectively (Table 1).

**Table 1:** Correlation between gelsolin expression and clinicopathological parameters of laryngeal squamous cell carcinoma.

Characteristics	Gelsolin expression			
	Nº of cases (%)	Negative (n, %)	Positive (n, %)	*p*-value
Age				0.794
≤ 60	73 (43.4%)	22 (30.1%)	51 (69.9%)	
> 60	95 (56.6%)	28 (29.4%)	67 (70.6%)	
Sex				0.937
Male	158 (5.9%)	47 (29.8%)	111 (70.2%)	
Female	10 (94.1%)	3 (30%)	7 (70%)	
Location				
Glottis	108 (64.2%)	35 (32.5%)	73 (67.5%)	0.623
Supraglottis	55 (32.9%)	14 (25.5)	41 (74.5%)	
Subglottis	5 (2.9%)	1 (20%)	4 (80%)	
Histological grade				0.031
Grade I	66 (39.2%)	25 (37.9%)	41 (62.1%)	
Grade II	82 (48.8%)	21 (25.7%)	61 (74.3%)	
Grade III	20 (11.9%)	4 (20%)	16 (80%)	
TNM stage				0.001
I–II	64 (38%)	29 (45.4%)	35 (54.6%)	
III–IV	104 (62%)	21 (20.2%)	83 (79.8%)	
Lymph node status				0.326
Negative	113 (67.3%)	34 (31.1%)	79 (69.9%)	
Positive	55 (32.7%)	16 (29.1%)	39 (70.9%)	
PNI				0.872
Yes	36 (21.4%)	9 (25.0%)	27 (75.0%)	
No	132 (78.6%)	41 (31.1)	91 (68.9%)	
LVI				0.016
Yes	53 (31.5%)	10 (24.6%)	43 (81.1)	
No	115 (68.5%)	40 (34.8%)	75 (65.2%)	
Loco-regional recurrence				0.018
Yes	60 (35.7%)	9 (15%)	51 (85%)	
No	108 (64.3)	41 (37.9%)	67 (62.1%)	

TNM, Tumor-Nodes-Metastases; PNI, Perineural Invasion; LVI, Lymphovascular Invasion.

The rate of immunohistochemical staining with gelsolin was found to be significantly higher in tumor tissues of patients with an advanced clinical stage than in patients with an early clinical stage (positive gelsolin expression was 79.8% for advanced-stage LSCC and 54.6% for early-stage LSCC, *p* < 0.05).

### Survival analysis

Recurrence was reported for 60 (35.7%) of 168 patients with LSCC during the follow-up period. The mean recurrence period was 28 ± 5.8 months, with a range of 3–43 months. A Kaplan–Meier analysis of survival showed that the mean period of recurrence was 30.4 months in patients with low gelsoline levels versus 17.9 months in patients with gelsoline overexpression, respectively (*p* = 0.002).

The median OS was 78.0 months, ranging from 1 to 120 months. The TNM stage (*p* = 0.031), lymph node status (*p* = 0.001), PNI (*p* = 0.041), and LVI (*p* = 0.002) were identified as factors associated with a significantly worse prognosis by univariate analysis. Multivariate analysis confirmed TNM stage, LVI, and positive gelsolin expression as independent prognostic factors indicating poorer outcomes, with *p*-values of 0.01, 0.021, 0.025 respectively.

**Figure 3:**
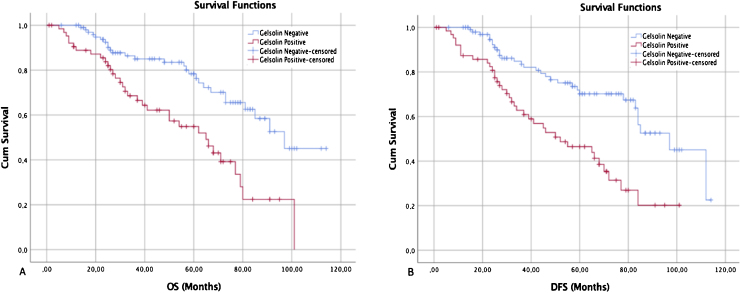
Kaplan–Meier survival curves showed that gelsolin expression was inversely correlated with both overall and disease-specific survival. (A) The overall survival curves of negative and positive gelsolin expression in LSCC patients (*p* < 0.05). (B) The disease-free survival curves of negative and positive gelsolin expression in LSCC patients (*p* < 0.05).

Regarding gelsolin, Kaplan–Meier survival curves revealed that LSCC patients with gelsolin-positive tumors had lower OS rates relative to patients with gelsolin-negative tumors (*p* = 0.004) (Fig. 3A). On the other hand, univariate Cox regression analysis for OS demonstrated that gelsolin immunopositivity predicted an increased risk of patient death (HR=3.28, 95% CI 1.67–7.47, *p* = 0.004). The prognostic significance of gelsolin was shown to be independent of clinicopathological parameters (age, sex, tumor location, histological location, TNM stage, lymph node status, PNI, and LVI) of the patient (HR = 2.63, 95% CI 1.27–6.67, *p* = 0.025) (Table 2).

**Table 2:** Univariate and multivariate analysis for overall survival in patients with laryngeal squamous cell carcinoma.

	Univariate analysis			Multivariate analysis		
	HR	95% CI	*p-*value	HR	95% CI	*p-*value
**Age**						
≤ 60	1.00	0.96–1.07	0.351	1.00	1.01–1.27	0.498
> 60	1.02			1.16		
**Sex**						
Female	1.00	0.42–2.61	0.857	1.00	0.49–2.12	0.630
Male	1.07			1.09		
**Location**						
Glottis-subglottis	1.00	1.02–1.93	0.492	1.00	1.12–2.14	0.412
Supraglottis	1.17			1.27		
**Histological grade**						
Grade I–II	1.00	0.95–2.02	0.212	1.00	1.05–1.85	0.087
Grade III	1.14			1.28		
**TNM stage**						
I–II	1.00	1.03–4.37	0.031	1.00	1.21–4.37	0.001
III–IV	2.15			2.55		
**Lymph node**						
Negative	1.00	1.41–5.4	0.001	1.00	1.10–4.1	0.069
Positive	2.08			1.38		
**PNI**						
No	1.00	0.89–4.53	0.041	1.00	0.83–2.94	0.093
Yes	1.65			1.15		
**LVI**						
No	1.00	1.23–5.48	0.002	1.00	1.15–4.92	0.021
Yes	2.69			2.38		
**Gelsolin expression**						
Negative	1.00	1.67–7.47	0.004	1.00	1.27–6.67	0.025
Positive	3.28			2.63		

TNM, Tumor-Nodes-Metastases; PNI, Perineural Invasion; LVI, Lymphovascular Invasion.

The median DFS was 68.0 months, ranging from 1 to 120 months (95% Confidence Interval, 95% CI 58.7–133.8) (Fig. 3B). Univariate analysis on DFS indicated that TNM stage, lymph node status, LVI, and gelsolin expression were significantly associated with poorer outcomes (*p* = 0.014, 0.001, 0.002, and 0.030 respectively), all factors were confirmed as independent prognostic factors for predicting poorer outcomes (*p* < 0.05) by multivariate analysis (Table 3).

**Table 3:** Univariate and multivariate analysis for disease-free survival in patients with laryngeal squamous cell carcinoma.

	Univariate analysis			Multivariate analysis		
	HR	95% CI	*p*-value	HR	95% CI	*p*-value
**Age**						
≤ 60	1.00	0.93–1.05	0.528	1.00	0.99–1.10	0.694
> 60	1.01			1.11		
**Sex**						
Female	1.00	0.47–2.94	0.941	1.00	0.41–2.19	0.672
Male	1.03			1.05		
**Location**						
Glottis-subglottis	1.00	1.13–8.71	0.081	1.00	1.61–8.41	0.073
Supraglottis	2.15			2.94		
**Histological grade**						
Grade I–II	1.00	0.95–3.61	0.134	1.00	1.25–6.62	0.064
Grade III	1.2			1.3		
**TNM stage**						
I–II	1.00	1.01–3.94	0.014	1.00	0.89–3.03	0.014
III–IV	2.03			1.83		
**Lymph node**						
Negative	1.00	1.65–6.15	0.001	1.00	1.44–6.33	0.015
Positive	3.23			3.01		
**PNI**						
No	1.00	0.43–2.45	0.806	1.00	0.49−2.19	0.749
Yes	1.29			1.03		
**LVI**						
No	1.00	0.89–3.8	0.002	1.00	0.86–4.14	0.047
Yes	1.97			2.31		
**Gelsolin expression**						
Negative	1.00	1.32–9.38	0.030	1.00	1.49–8.12	0.020
Positive	2.86			3.51		

TNM, Tumor-Nodes-Metastases; PNI, Perineural Invasion; LVI, Lymphovascular Invasion.

## Discussion

Currently, proven clinical parameters, such as TNM classification and histopathological grading, are insufficient to accurately predict the exact prognosis of LSCC patients.^16^ Biological markers are widely used in head and neck cancer to enhance the prediction of tumor aggression. Her-2 and p53 proteins are well-established molecules presently used to assess the prognosis of patients with LSCC.^17^, ^18^ While these biomarkers can identify poor prognosis, additional cellular research is still required to establish new innovative treatment options. With the discovery of novel biomarkers, more accurate treatment modalities can be offered for LSCC patients and may lead to longer survival rates.^19^ In this research, we studied whether the expression level of gelsolin could serve as a potential biological marker for prognosis and survival of LSCC.

Gelsolin participates in the reorganization of actin cytoskeleton that lead to regulation of apoptosis and cell motility.^8^ Mitochondria-mediated apoptosis is well known as being stimulated by the loss of membrane potential and the release of proapoptotic cytochrome c, which activates caspase and induces nuclear fragmentation. Gelsolin overexpression prevents mitochondrial membrane potential loss and inhibits apoptosis.^20^ Kusana et al. (2016) reported that gelsolin overexpression counteracts mitochondrial membrane potential loss and cytochrome c release, by blocking caspase-3, -8 and -9 activation in cancer cells, thus inhibiting apoptosis.^21^

To date, gelsolin expression in LSCC has not been studied; therefore, in this research, the potential impact of gelsolin expression on LSCC was studied and analyzed. Gelsolin expression status was found to have a statistically significant relationship in the prognosis of laryngeal cancer. However, results of this research cannot be directly compared with current literature, due to lack of similar investigations into LSCC.

Gelsolin protein might be considered as a promising molecular marker for prognostic evaluation in human cancer. It remains unclear whether the increased or decreased expression of gelsolin is associated with adverse prognosis in human neoplasms, which is possibly due to the nuanced and multifactorial nature of tumorigenesis.

Gelsolin protein has been observed to be elevated in a considerable number of solid tumors. Several studies have demonstrated that increased expression of gelsolin is related to lower survival rates among patients with human prostate cancer, lung cancer, and oral cavity cancer.^9^, ^10^, ^11^, ^15^, ^22^ Moreover, a negative expression of gelsolin in patients with colon carcinoma closely associated with longer survival rates.^13^ However, the downregulation of gelsolin in renal clear cell carcinoma has been reported to be indicative of worse prognosis.^23^ Although gelsolin can apparently have opposite actions depending on tumor type, detailed analysis of its expression status in different tumor sites are crucial for understanding its impact on carcinogenesis.

Our data revealed that overexpression of the gelsolin protein appears significantly correlated with unfavorable OS and DFS in patients with LSCC. Zhu et al. (2012) noticed that longer OS and DFS was significantly associated with the downregulation of gelsolin protein for squamous cell lung cancer, which correlates with our findings and results reported by Yang et al. (2004).^9^, ^24^ It is possible to infer that gelsolin immunopositivity could be a guide to identify LSCC patients with shorter OS and DFS. It is also possible to expect similar effects of gelsolin overexpression in other types of head and neck squamous cell cancers.

In the literature, only two articles have reported the effect of gelsolin on head and neck cancers. Shieh et al. (2006) studied the effects of gelsolin expression on oral carcinogenesis and found a significant relationship between gelsolin overexpression and shorter DFS. Additionally, increased gelsolin expression, from precancerous lesions to cancer cells, has been reported.^11^ According to Wang (2014), head and neck cancer cell lines with higher endogenous gelsolin expression were more cisplatin resistant than those with lower gelsolin expression.^22^ Our study supports these articles, revealing a relationship between poor prognosis and gelsolin overexpression, but gelsolin effect in the process of cancer transformation from precancerous lesions in LSCC has still not been studied.

Our analysis showed a strong association between histopathological tumor grade and expression of gelsolin. In patients with poorly differentiated LSCC, gelsolin immunopositivity was higher than in patients who had moderate or well differentiated tumors. A similar result has been reported in non-small cell lung carcinoma, where a high gelsolin expression was associated with increased tumor grade.^9^ We also observed in this study that increased expression of gelsoline was correlated with higher LVI rates. Although the mechanism behind the LVI has not been clearly proven, it may represent a tumor microenvironment that predicts aggressive tumor behavior. In particular, survival rates of LSCC patients were significantly lower if LVI was observed in pathological specimens.

In addition, we have found a correlation between expression of gelsolin and tumor stage in LSCC. Expression of gelsolin was higher in patients with advanced-stage LSCC relative to those with early-stage LSCC. This may suggest that in the early stages of laryngeal carcinogenesis the pattern of gelsoline expression is of little relevance. However, some authors found no relationship between tumor stage and gelsolin expression.^13^

Increased motility ability is crucial for tumor cells to invade and metastasize.^25^ Various studies reported that increased motility functions of cells are related to higher rates of tumor invasion and reduced survival time.^26^, ^27^ In some animal models, the motility regulating role of gelsolin has been observed.^28^, ^29^ In 229 patients with lung carcinoma, Shieh et al. (1999) found a strong inverse relationship between the level of gelsolin expression and survival. So that he hypothesized that the impact of gelsolin on cell motility may be the explanation for the poor prognostic role of high gelsolin expression.^14^ Consequently, tumor cells might upregulate gelsolin to increase movement capability, as achieving highly mobile behavior is considered a prerequisite for the spread of a tumor cell to adjacent tissues and distant metastasis. In our study, a significant relationship was found between gelsolin overexpression and locoregional recurrence: one of the most important reasons for this phenomenon may be the enhancing effect of gelsolin on motility.

Although a relationship between gelsolin and LSCC was clearly revealed in our study, several limitations were associated with this article. Because of IHC evaluation was based on a subjective scoring method, results depend on the experience of the person making the assessment. In addition, it remains unclear why the increased expression of gelsolin was closely associated with adverse prognosis in patients with LSCC. Further cellular studies are required to validate prognostic value of gelsolin against other parameters in a large group of patients.

In conclusion, gelsolin immunopositivity was significantly associated with poorer prognosis in patients with LSCC. Moreover, we conclude that gelsolin expression could be regarded as a novel independent prognostic biomarker for OS and DFS in patients with LSCC. Therefore, our results may provide evidence that gelsolin may contribute to the development, progression, and prognosis of LSCC. This study also reveals that new therapeutic agents targeting gelsolin protein may be useful to treat laryngeal cancer more effectively. Further evaluation of gelsolin effects on LSCC is required to validate our results.

All authors declare no conflict of interest, and no external funding was obtained for this study.

## Conflicts of interest

The authors declare no conflicts of interest.
